# Precisely Control Relationship between Sulfur Vacancy and H Absorption for Boosting Hydrogen Evolution Reaction

**DOI:** 10.1007/s40820-023-01291-3

**Published:** 2024-01-02

**Authors:** Jing Jin, Xinyao Wang, Yang Hu, Zhuang Zhang, Hongbo Liu, Jie Yin, Pinxian Xi

**Affiliations:** College of Chemistry and Chemical Engineering, Frontiers Science Center for Rare Isotopes, State Key Laboratory of Applied Organic Chemistry, Lanzhou University, Lanzhou, 730000 People’s Republic of China

**Keywords:** Hydrogen evolution reaction, S vacancies, Nanosheet, H, Adsorption

## Abstract

Effective and robust catalyst is the core of water splitting to produce hydrogen. Here, we report an anionic etching method to tailor the sulfur vacancy (V_S_) of NiS_2_ to further enhance the electrocatalytic performance for hydrogen evolution reaction (HER). With the V_S_ concentration change from 2.4% to 8.5%, the H* adsorption strength on S sites changed and NiS_2_-V_S_ 5.9% shows the most optimized H* adsorption for HER with an ultralow onset potential (68 mV) and has long-term stability for 100 h in 1 M KOH media. In situ attenuated-total-reflection Fourier transform infrared spectroscopy (ATR-FTIRS) measurements are usually used to monitor the adsorption of intermediates. The S- H* peak of the NiS_2_-V_S_ 5.9% appears at a very low voltage, which is favorable for the HER in alkaline media. Density functional theory calculations also demonstrate the NiS_2_-V_S_ 5.9% has the optimal |ΔG_H*_| of 0.17 eV. This work offers a simple and promising pathway to enhance catalytic activity via precise vacancies strategy.

## Highlights

- The Ar plasma etching strategy was introduced to homogeneously distributed S-vacancies (VS) into the NiS_2_ nanosheets (NiS_2_-VS).
- Build the relationship between sulfur vacancy and H absorption and find that NiS_2_-VS 5.9% performs outstanding hydrogen evolution reaction performance and remarkable stability.

## Graphical abstract

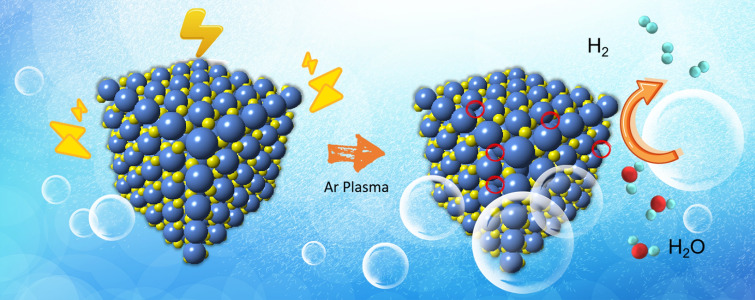

## Introduction

Hydrogen energy usually be regarded as the most promising clean energy source for development at present [1–3]. And electrochemical hydrogen evolution reaction (HER) is now considered one of the most effective and clean method to produce H_2_, as a promising candidate for sustainable development. In addition, industry tends to produce hydrogen in alkaline media due to their low cost, easy to manufacture and good performance [4–7]. However, there are still challenges in alkaline media, and it is difficult to generate H* through the HER. In general, the appropriate H_2_O adsorption and dissociation energy is certainly one deciding factor for this process [8, 9]. Crucial to addressing this challenge need to find high catalytic activity and good stability catalysts for HER. To date, Pt-based compounds are the most effective catalysts for HER, but their large-scale application is limited by high price, scarcity and poor stability [10–15]. Therefore, designing effective, inexpensive and stable non-noble metal catalysts for HER will be the focus of future research.

Transition metal sulfides (TMS) usually be recognized as promising noble-metal-free electrocatalysts for HER due to the inherent high electrical transport and ideal atomic arrangement [16–21]. Moreover, through a series of means which contain doping heteroatoms [22], controlling crystal face [23], engineering defects [24, 25], strains, etc*.* [26]. Therefore, using these microstructure modulations can effectively modify the atomic structures of catalyst. Moreover, the chemical environment and electronic structure of TMS will be tuned by the vacancies control strategy, the local reaction environment of the catalysts also plays a crucial role in electrode processes, thus, to optimize the adsorption of reaction intermediate and accelerate the HER kinetics. However, developing a precise vacancy modulation approach remains a challenge that requires more in-depth studies.

Herein, we report a facile Ar plasma etching strategy approach is proposed to introduce homogeneously distributed S-vacancies (Vs) onto the NiS_2_ nanosheet surface. And the S vacancy concentration can be precise tuning by varying the etching time. The obtained optimized material of NiS_2_-Vs is 5.9% which means the NiS_2_ nanosheet with S-vacancies concentration of 5.9% under the Ar plasma etching for 3 min. This catalyst has the optimal S-vacancy state. Using the in situ ATR-FTIRS and density functional theory (DFT), the optimized H* adsorption strength is proven. Thus, this catalyst performs a superior HER performance. In strong alkaline electrolytes (for example, 1.0 M KOH), this catalyst shows an overpotential of only 108 mV at a current density of 10 mA cm^−2^ and can maintain long-term stability up to 100 h, which are superior to those of the recently reported breakthrough HER catalysts. And this NiS_2_ structure will occur the phase evolution under HER conditions. We conclude that precisely control of the S-vacancies concentration will open significant opportunities for rational design of electrocatalysts from earth-abundant and stable materials.

## Experimental and Calculation

### Synthesis

#### Synthesis of NiLDH Precursor

Using nickel (II) nitrate hexahydrate Ni(NO_3_)_2_·6H_2_O, 0.1 mol L^−1^, 60 mL as the reaction solution. Then, the three-electrode system was used to electrochemical deposition NiLDH. In detail, the Ag/AgCl electrode was used as a reference electrode, Pt net was employed as an auxiliary electrode, and the working electrodes were carbon cloth (CC). The NiLDH can be obtained on the working electrode under constant potential of − 1.0 V for 60 min. The precipitate was rinsed with water and ethanol for more than three times and dried under vacuum at 50 °C for 4 h.

#### Synthesis of NiS_2_ Nanosheets

In generally, 300 mg sulfur powder and two-piece of the NiLDH arrays on the carbon cloth (about 2 × 3 cm^2^) were placed in two crucibles, which are 20 cm apart in the tube. Then, the tube was heated to 300 °C with a rate of 10 °C min^−1^ for 2 h under the flowing Ar atmosphere. Finally, the system was cooled under a flowing Ar atmosphere and NiS_2_ nanosheets would be obtained.

#### Synthesis of P-NiS_2_ Nanosheets

In generally, the NiS_2_ nanosheets were using plasma etching technology, at different etching time, can be obtained different S vacancies concentration NiS_2_ nanosheets.

### Materials Characterizations

X-ray diffraction (XRD) experiments were conducted 2 from 10° to 90° on an X'Pert ProX-ray diffractometer with Cu Ka radiation (*λ* = 0.1542 nm) under a voltage of 40 kV and a current of 40 mA. Sample compositions were determined by ICP-AES (HITACHI P-4010, Japan). Field-emission scanning electron microscopy (FESEM, Zeiss) at an acceleration voltage of 5 kV. All samples were coated with a thin layer of gold prior to FESEM observations. Transmission electron microscopy (TEM) and high-resolution transmission electron microscopy (HRTEM) observations were performed under an acceleration voltage of 200 kV with a JEOL JEM 2100 TEM. Atomic-scale STEM images were recorded on a probe aberration-corrected STEM (Cubed Titan G2 60-300, FEI, USA) operated at 300 kV. X-ray photoelectron spectroscopy (XPS) analyses were made with a VG ESCALAB 220I-XL device. All XPS spectra were corrected using C 1*s* line at 284.6 eV. The absorption spectra of M (M = S, Ni) L/K-edge and were collected in transmission mode using a Si (111) double-crystal monochromator at the BLW141 station of the Shanghai Synchrotron Radiation Facility (SSRF).

### Electrochemical Test for HER

Electrochemical measurements were carried out at room temperature using the three-electrode system directly connected to a CHI 760 E Electrochemical Workstation (CHI Instruments, Shanghai Chenhua Instrument Corp., China). Saturated Hg/HgO (in a saturated 1.0 M KOH solution) was employed as a reference electrode, the corresponding catalysts were used as working electrode, and a graphite rod was employed as a counter electrode. The potentials were referenced to the RHE (*E*_RHE_ = *E*_Hg/HgO_ + 0.098 V + 0.0591pH V). Also, a resistance test was made and the iR compensation was applied by using the CHI software.

### Theoretical Calculations

All spin-polarized calculations are performed in the framework of the density functional theory with the projector augmented plane-wave method, as implemented in the Vienna ab initio simulation package [27]. The generalized gradient approximation proposed by RPBE is selected for the exchange–correlation potential [28]. The DFT-D3 method was used to describe the van der Waals interactions between intermediate and catalyst [29]. The DFT + U method was used to optimize the geometric and electronic structures, where U value was 3.40 for Ni. The cut-off energy for plane wave is set to 450 eV. The energy criterion is set to 10^−4^ eV in iterative solution of the Kohn–Sham equation. A vacuum layer of 15 Å is added perpendicular to the sheet to avoid artificial interaction between periodic images. The Brillouin zone integration is performed using a 3 × 3 × 1 k-mesh. All the structures are relaxed until the residual forces on the atoms have declined to less than 0.02 eV Å^−1^.

The adsorption energy (Δ*E*_ads_) and free energy changes (Δ*G*) of reaction intermediates could be calculated by the following:

1 $$\Delta {\text{E}}_{{{\text{ads}}}} = {\text{ E}}_{{{\text{ads}}}}^{*} - {\text{E}}_{{{\text{ads}}}} - {\text{E}}_{{{\text{slab}}}}$$

2 $$\Delta {\text{G }} = \, \Delta {\text{E }} + \, \Delta {\text{E}}_{{{\text{ZPE}}}} - {\text{T}}\Delta {\text{S}}$$

where *E*_ads_ and *E*_slab_ are the energy of H + and substrate, respectively. The Δ*E* is the adsorption energy on the cluster surface from DFT calculations. The Δ*E*_ZPE_ and Δ*S* are the difference for the zero-point energy and entropy. The zero-point energy and entropy are calculated at the standard conditions corresponding to the pressure of 10,1325 Pa (~ 1 bar) of H_2_ at the temperature of 298.15  K.

## Results and Discussion

### Catalyst Design and Structural Characterization

**Fig. 1:**
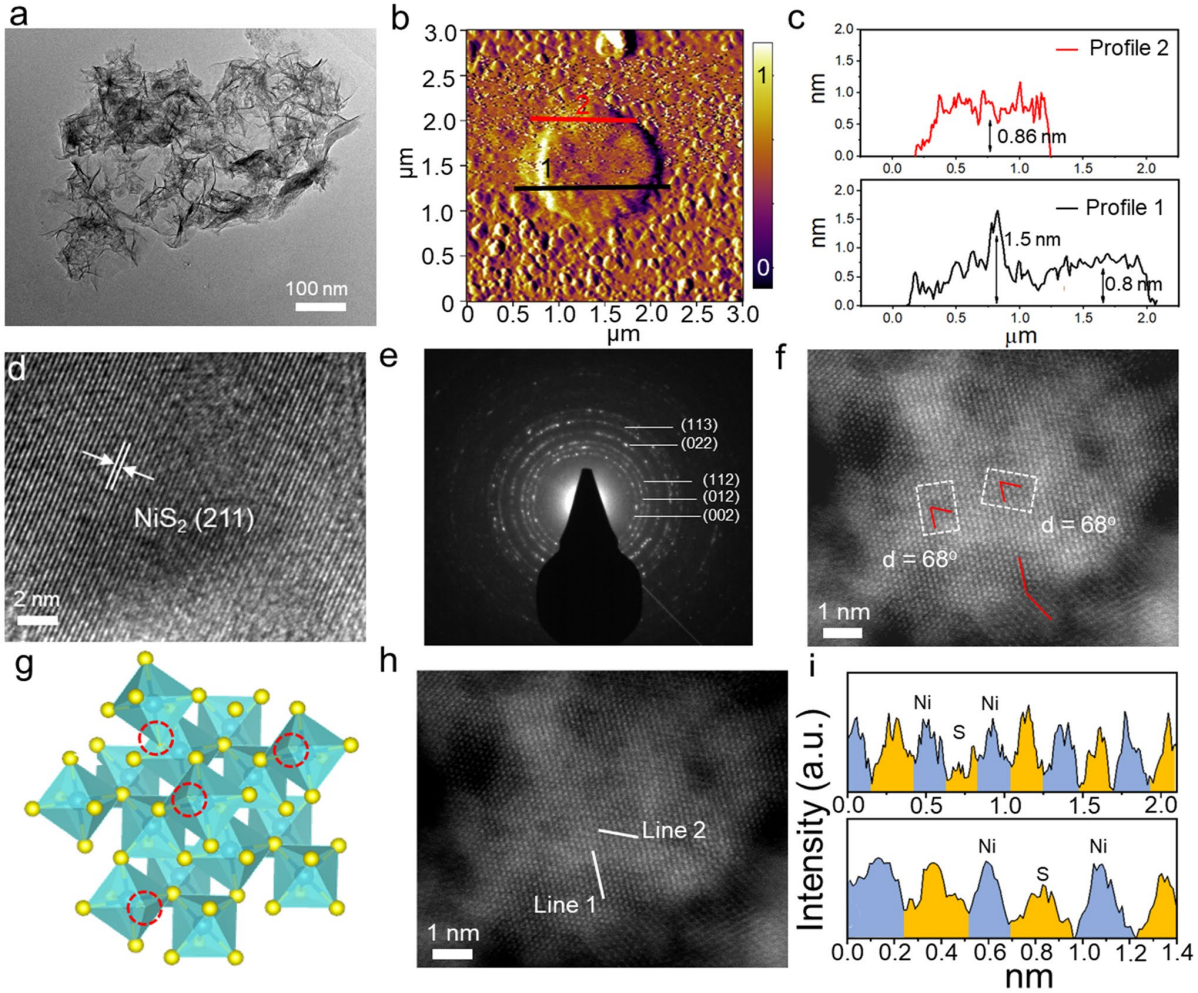
**a** TEM images of NiS_2_-Vs 5.9% NSs; **b** AFM image of the NiS_2_-Vs 5.9% NSs; **c** The corresponding height profiles obtained from two different line scans of 1, 2 in (b). **d** HRTEM images of NiS_2_-Vs 5.9% NSs;** e** SAED image of NiS_2_-Vs 5.9% NSs; **f** HAADF-STEM image of NiS_2_-Vs 5.9% NSs, the insert white squares show the lattice deformation of S vacancies introduced; **g** Crystal structure of NiS_2_-Vs 5.9% NSs; **h** HAADF-STEM image of S vacancies in NiS_2_-Vs 5.9% NSs; **i** Intensity profile of line 1 and 2 areas

We developed the Ni-LDH nanosheets precursor via electrochemical deposition, then the NiS_2_ NSs are obtained through the calcination of the corresponding LDH under sulfur stream at 300 °C for 2 h, and finally, we introduced the *S*-vacancies on NiS_2_ NSs by Ar plasma etching strategy and adjusting *S*-vacancy concentrations via controlling etching time. In general, the TEM is applied to characterize the morphology of the synthesized materials, as shown in Fig. 1a, the NiS_2_-Vs 5.9% performs a uniform ultra-thin nanosheet structure. From Fig. 1b, combined with the atomic force microscopy (AFM), we could evaluate the thickness of the NiS_2_-Vs 5.9% NSs, and the results further confirm the 2D morphology of these NiS_2_ NSs. The AFM height profiles of the NiS_2_-Vs 5.9% NSs (Fig. 1c) revealed that the nanosheets presented a uniform height of approximately 0.8 nm. Additionally, the HRTEM could confirm the fine structure of NiS_2_-Vs 5.9% NSs. From Fig. 1d, the HRTEM image of NiS_2_-Vs 5.9% NSs displays the lattice space of 0.23 nm that matches with the (211) plane of NiS_2_. The introduced S vacancies did not cause change in the overall structure with NiS_2_ NSs (Figs. S1 and S2). The selected-area electron diffraction (SAED) also confirms the NiS_2_-Vs 5.9% NSs maintain the NiS_2_ crystal structure after Ar plasma (Fig. 1e). At the same time, in order to further confirm the successful introduction of S vacancies in NiS_2_-Vs 5.9% NSs, the high-angle annular dark field aberration-corrected scanning TEM (HAADF-STEM) was used and clearly observed the atomic arrangement of NiS_2_-Vs 5.9% NSs (Fig. 1f), which shows the S vacancies introduced caused lattice distortion in local areas. In addition, the introduced S vacancies in the NiS_2_-Vs 5.9% NSs are examined by HAADF-STEM shown in Fig. 1h, g. By analyzing the atom arrangement of lines 1 and 2, indicating the presence of S vacancies in the NiS_2_-Vs 5.9% NSs, the corresponding line intensity profiles in Fig. 1i can directly display the different periodic atom arrangements of line 1 and 2 and reconfirm the introduced S vacancies in the NiS_2_-Vs 5.9% NSs.

**Fig. 2:**
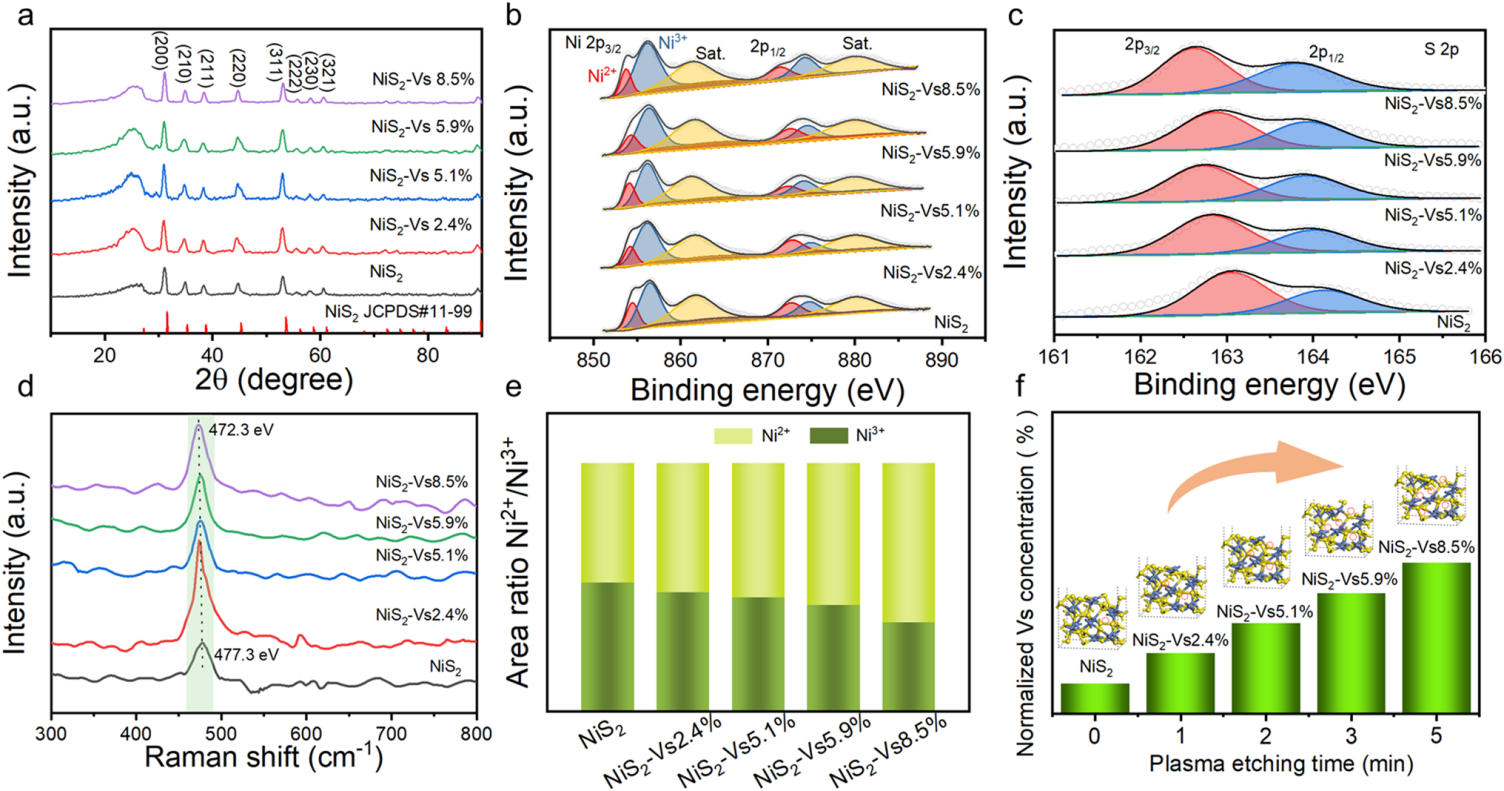
**a** XRD patterns of the NiS_2_ nanosheets with a series of S vacancies concentrations; **b, c** Ni 2*p* spectra and S 2*p* spectra of XPS for the NiS_2_ nanosheets with a series of S vacancies concentrations; **d** Raman spectra of the NiS_2_ nanosheets with a series of S vacancies concentrations; **e** The area ratio Ni^2+^/Ni^3+^ of the NiS_2_ nanosheets with a series of S vacancies concentrations; **f** Histogram of the calculated S vacancies concentrations

The electronic and coordination structures of NiS_2_ which using Ar plasma etching were further investigated using various spectroscopy methods. From Fig. 2a, these NiS_2_ NSs which under different etching time display the same XRD pattern of cubic NiS_2_ with a space group of Pa3 (JCPDS No. 11 − 99, *a* = *b* = *c* = 5.670 Å), has same result with the HRTEM image. In addition, combined with XPS could exploring the impact of introducing sulfur defects on the electronic environment of materials. As shown in Fig. 2b, the XPS of Ni 2*p*, which contained with two spin–orbit doublets characteristic of Ni^2+^, Ni^3+^ and two shakeup satellites (identified as “Sat”). By analyzing the peak area of two spin–orbit doublets characteristic of Ni^2+^ and Ni^3+^, the introduced sulfur vacancies by Ar plasma etching could cause the electronic environment to change of NiS_2_, the proportion of Ni^2+^/Ni^3+^ increase with growth of etching time, indicates that as the etching time increases, more sulfur vacancies will be introduced and resulting in decrease the metal valence state in NiS_2_ (Fig. 2e). Meanwhile, the electronic environment change of Ni site could optimize the adsorption and dissociation of water, which were advantageous to HER. Tailoring sulfur vacancies concentration plays important role of electronic structure change; thus, we need to clarify the different sulfur vacancies concentrations which caused by Ar plasma etching. As depicted in Fig. 2c, S 2*p* XPS spectra have two peaks about S 2*p*_1/2_ peak and S 2*p*_3/2_ peak, according to the previous report, and the S 2*p*_1/2_ peak corresponding to low coordination sulfur is related to sulfur vacancies [30]. Thus, we have estimated the sulfur vacancy concentration of all NiS_2_ which using Ar plasma etching. As shown in Fig. 2f, with etching time extension the sulfur vacancy concentration also gradually increases, and this is consistent with the previous conclusion. Figure 2d shows the Raman spectra of the NiS_2_ NSs with different S vacancies concentrations; from the detailed vibrational modes of the Raman spectra, we can observe the characteristic peaks of NiS_2_ and with the increase of S vacancies, and this characteristic peaks gradually occur red shift.

### Electrocatalytic Performance

**Fig. 3:**
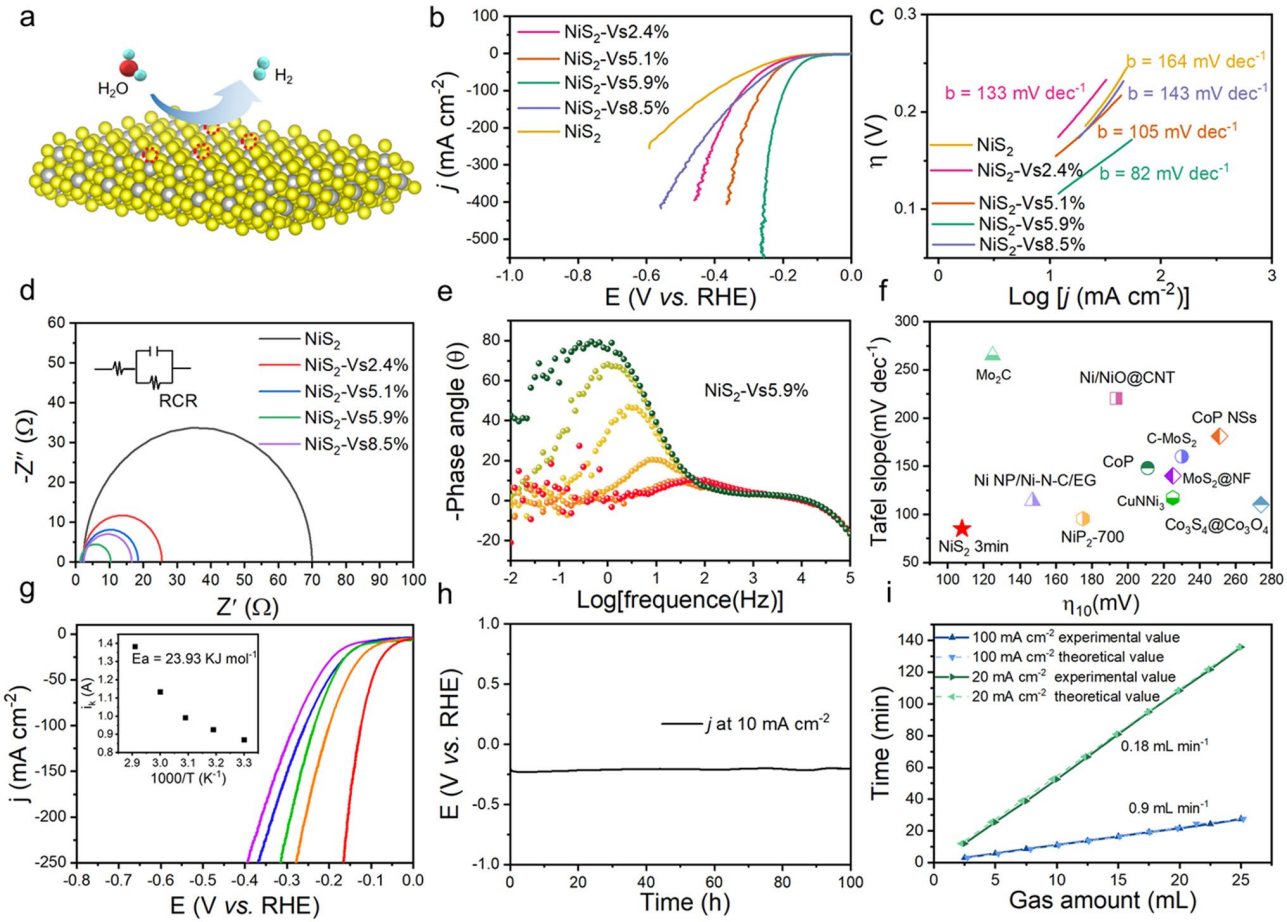
**a** Schematic diagram of hydrogen evolution of the NiS_2_ NSs with S vacancies concentrations. **b** IR-corrected LSV curves, **c** Tafel plots and **d** EIS curves of the NiS_2_ nanosheets with a series of S vacancies concentrations; **e** Bode phase plots of NiS_2_-Vs 5.9%; **f** Comparison of merit with respect to both kinetics (Tafel slope) and activity; **g** LSV curves of NiS_2_-Vs 5.9% NSs for HER at different temperatures. The inset in **g** shows Arrhenius plot of the kinetic current; **h** Chronoamperometric response of NiS_2_-Vs 5.9% NSs for HER at 10 mA cm^−2^; **i** Experimentally and theoretical measured H_2_ amounts based on NiS_2_-Vs 5.9% NSs

The HER catalytic activity of these materials which contain the NiS_2_ at different times Ar plasma etching was assessed as the working electrode and evaluated by linear scan voltammogram (LSV) in N_2_-saturated 1.0 M KOH electrolyte (pH = 14) in a three-electrode cell (Fig. 3a). Figure 3b shows the HER performance of these catalysts. As we think, NiS_2_-Vs 5.9% shows excellent HER performance which has ultra-low overpotentials, when the current density at 10 mA cm^−2^, the overpotential just 108 mV, it’s much better than those contrast materials which contain NiS_2_ (182 mV), NiS_2_-Vs 2.4% (165 mV), NiS_2_-Vs 5.1% (150 mV) and NiS_2_-Vs 8.5% (146 mV). Additionally, the Tafel plots (log j ~ *η*) also play a significant role in electrocatalytic can further measured to confirm the HER kinetic rate of those catalysts. As shown in Fig. 3c, NiS_2_-Vs 5.9% has the smallest Tafel role (82 mV dec^−1^) indicating the fastest HER kinetics. Apart from that the electrochemical impedance spectroscopy (EIS) analysis could further investigate the electrode kinetics during the HER process. From Fig. 3d, e shows the Nyquist plots of these electrodes, the NiS_2_-Vs 5.9% with low solution resistance (*R*_s_) of 1.25 Ohm and charge-transfer resistance (*R*_ct_) of 4.5 Ohm (Table S1), was obviously lower than other contrast materials. Meanwhile, EIS also could be used to elucidate the redox processes at different potentials, the NiS_2_-Vs 5.9% phase angle maximum in the Bode plot moved fastest to lower values (Fig. S3), which further confirm the NiS_2_-Vs 5.9% has fastest HER kinetics. Among some representative HER catalysts reported, the NiS_2_-Vs 5.9% with present state-of-the-art alkaline HER catalysts (Fig. 3f and Table S2) [31–42]. Furthermore, to assess the kinetic barriers involved in HER, we studied the effect of temperature on the performance of NiS_2_ NSs in alkaline media (Figs. 3g and S4–S8). Meanwhile, we found the *E*_app_ value which calculated from the Arrhenius equation will reach its maximum around the respective catalytic OER onset potential, the NiS_2_-Vs 5.9% demonstrates the low apparent barrier value of 23.9 kJ mol^−1^ at a lower onset potential compared to other catalysts, suggests has the good OER catalyst activity. Additionally, the stability is one of the important criteria to measure the performance of catalyst. From Fig. 3h, NiS_2_-Vs 5.9% performs perfect stability at current densities 10 mA cm^−2^ for 100 h in alkaline media with a negligible overpotential change. At the same time, we used the drainage method recorded the H_2_ production rate; from Fig. 3i, the hydrogen evolution rate could reach 0.9 mL min^-1^ at current densities 100 mA cm^−2^.

### Mechanism Analysis of the Enhanced Catalytic Performance

**Fig. 4:**
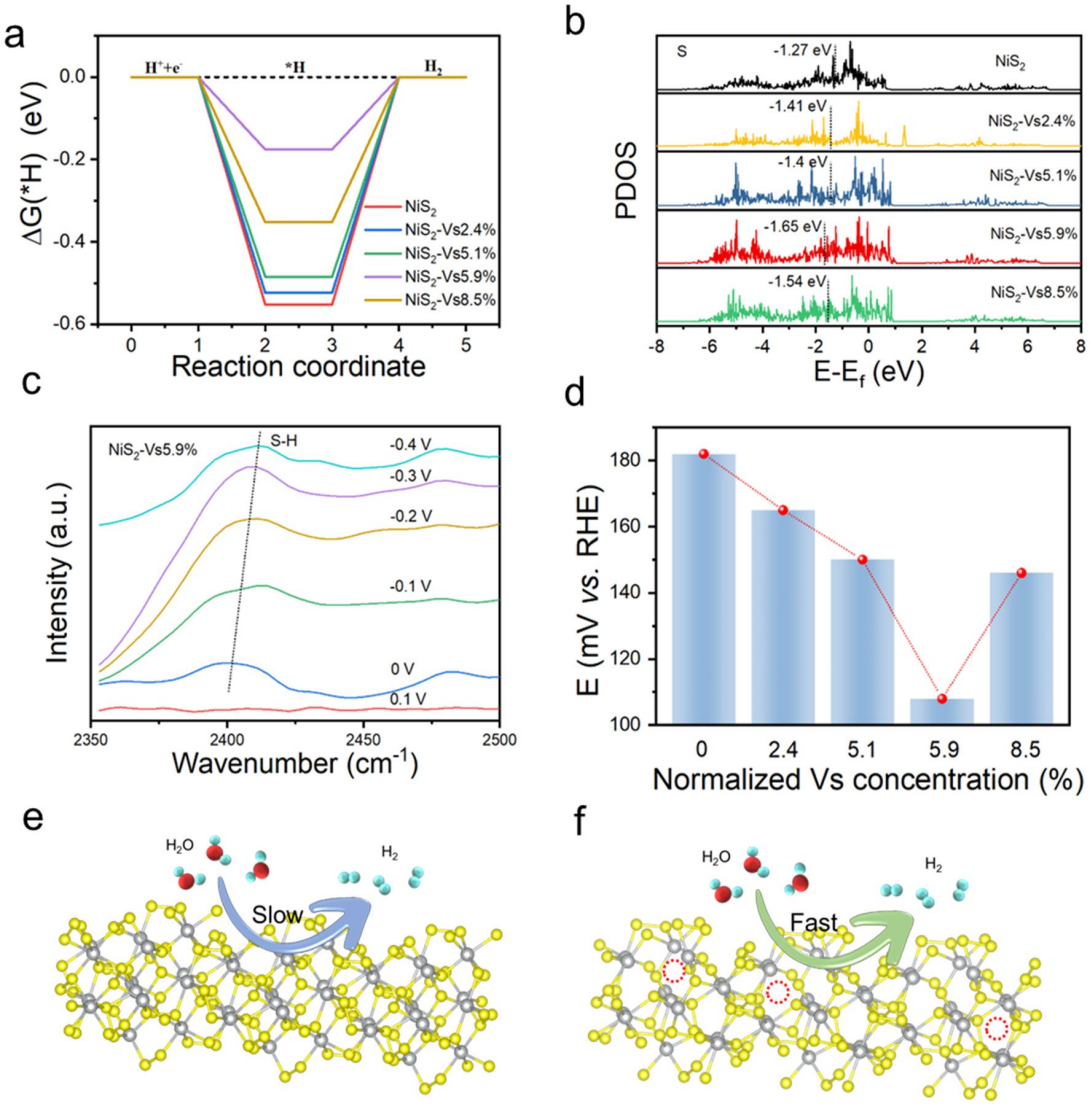
**a, b** PDOS and ΔG_H*_ of the NiS_2_ nanosheets with a series of S vacancies concentrations; **c** *H bands recorded on S sites in NiS_2_-Vs 5.9%; **d** Catalytic activity of different S vacancies concentrations; **e, f** Scheme of NiS_2_ nanosheets catalyst under the HER process

To explore the synergistic effect of the S-vacancy concentration and distribution on the HER properties, we systematically performed DFT calculations of Δ*G*_H*_, which is a well-known descriptor for theoretically predicting catalytic activity. As shown in Figs. 4a, b and S9, the Δ*G*_H*_ of NiS_2_-Vs 5.9% is closest to 0, and we all know the free energy of H* close to zero is considered to show high activity, this result is also consistent with previous experiments. And the density of states is further calculated to understand the intrinsic activity. Apart from that, we utilize the in situ spectroscopy to further reveal the origin of the activity of H atom adsorption on the active site. As shown in Figs. 4c and S10, the in situ ATR-FTIRS measurements were conducted to monitor the possible adsorption site and changes of binding energies of the reaction intermediates, the S–H peak appears at a low voltage, which indicate the introduced sulfur vacancies optimizes the H* adsorption on S sites, also resulting in the extremely enhanced HER activity for NiS_2_-Vs 5.9% NSs. In addition, the relationship of catalytic activity and S vacancies concentrations as shown in Fig. 4d. All in all, these results as shown in the schematic diagram (Fig. 4e, f), the catalyst which is treated by Ar plasma etching will introduced S vacanies and conducive to boost HER.

### Dynamic Phase Reconstruction

**Fig. 5:**
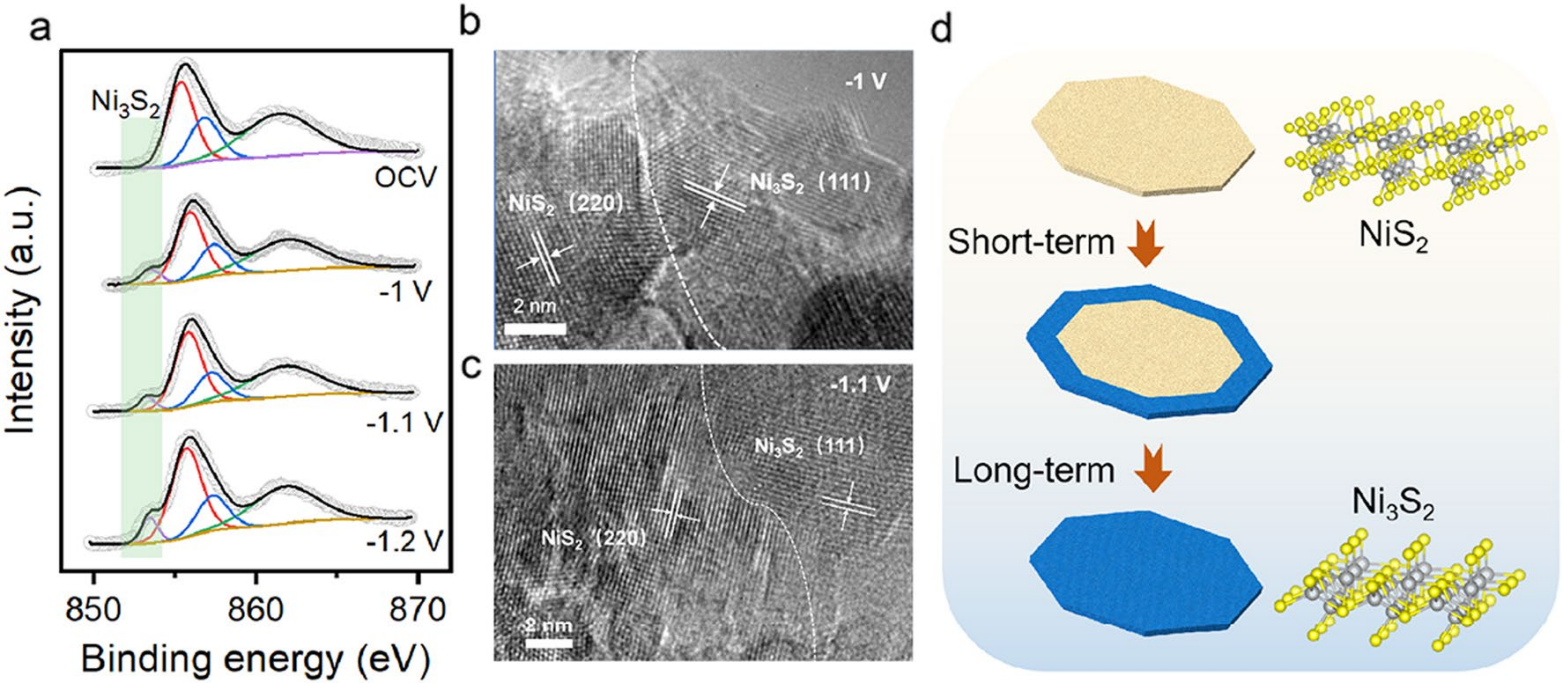
**a** Ni 2*p* XPS spectrum of NiS_2_-Vs 5.9% NSs under the HER process; **b, c** HRTEM images of NiS_2_-Vs 5.9% NSs at − 1 V and − 1.1 V, respectively; **d** Schematic illustration of the dynamic reconstruction of NiS_2_ NSs during HER process

After the HER catalytic test, we re-examined the crystal structure of NiS_2_-Vs 5.9%. According to the ex-XPS test, we studied the dynamic reconstruction of nickel sulfides surface during the HER process. As shown in Fig. 5a, we found that when the applied reduction potentials gradually increased, the corresponding characteristic peaks of Ni_3_S_2_ could be identified. And from the in situ Raman spectrum, we could observed the characteristic peak of Ni_3_S_2_, further prove have formed Ni_3_S_2_ structure in the HER process (Fig. S11). At the same time, combined with the HRTEM images (Fig. 5b, c),  the NiS_2_-Vs 5.9% NSs occur surface reconstruction and form the (111) plane of Ni_3_S_2_ after 30 min operation under the − 1 V and − 1.1 V condition, respectively. In general, just as the schematic diagram (Fig. 5d), the NiS_2_ structure will occur surface reconstruction into the Ni_3_S_2_ layer during the HER process. For NiS_2_ nanocrystal, the phase evolution and valence evolution in the whole HER process were revealed through a series of spectroscopy and microscope techniques, which can directly observe the reconstruction process and establish the dynamic structure activity correlation of its life.

## Conclusions

In conclusion, we have successfully developed an Ar plasma etching strategy to precise control the different concentrations of S vacancies in NiS_2_ NSs, and the catalyst NiS_2_-Vs 5.9% has perfected HER activity which has ultra-low overpotentials and good stability and can maintain 100 h in alkaline solution. The experimental and theoretical results confirm that the introduced S vacancies tuned the chemical environment and electronic structure of NiS_2_ and thus successfully optimized the adsorption energy of proton H. And further study indicated the phase evolution of NiS_2_ structure convert to the Ni_3_S_2_ structrue under HER process. This work will facilitate the development of novel high-performance electrocatalysts by vacancies strategy and provide insights into how to manipulate the electronic structure and chemical environment of metal sulfide catalysts to enhance the activity in a broad range of applications including HER.

## Data Availability

The data that support the findings of this study are available from the corresponding author upon request.
